# Metabolic control and bone health in adolescents with type 1 diabetes

**DOI:** 10.1186/1687-9856-2011-13

**Published:** 2011-10-26

**Authors:** Jill H Simmons, Miranda Raines, Kathryn D Ness, Randon Hall, Tebeb Gebretsadik, Subburaman Mohan, Anna Spagnoli

**Affiliations:** 1 Department of Pediatrics, Division of Endocrinology and Diabetes, Vanderbilt Children's Hospital, Nashville, TN, USA; 2 Department of Pediatrics, Division of Pediatric Endocrinology, Seattle Children's Hospital, Seattle, WA, USA; 3 Department of Biostatistics, Vanderbilt University Medical Center, Nashville, TN, USA; 4 Musculoskeletal Disease Center, Jerry L Pettis VA Medical Center, and Departments of Medicine and Biochemistry, Loma Linda University, Loma Linda, CA, USA; 5 Department of Pediatrics, Division of Pediatric Endocrinology, University of North Carolina at Chapel Hill, Chapel Hill, NC, USA

**Keywords:** bone mineral density, intact parathyroid hormone, insulin-like growth factor, type 1 diabetes, adolescent

## Abstract

**Background:**

Adults with type 1 diabetes (T1D) have decreased bone mineral density (BMD) and increased fracture risk, yet the etiologies remain elusive. Early detection of derangements in bone biomarkers during adolescence could lead to timely recognition. In adolescents with T1D, we evaluated the relationships between metabolic control, BMD, and bone anabolic and turnover markers.

**Methods:**

Cross-sectional study of 57 adolescent subjects with T1D who had HbA1c consistently ≥ 9% (Poor Control, PC n = 27) or < 9% (Favorable Control, FC n = 30) for two years prior to enrollment. Subjects had T1DM for at least three years and were without diabetes complications, known celiac disease, or other chronic diseases.

**Results:**

There were no differences between HbA1c groups in BMD, components of the IGF system, or 25-hydroxyvitamin D status. The prevalence of 25-hydroxyvitamin D abnormalities was similar to that seen in the general adolescent population. Few patients met the recommended dietary allowance (RDA) for vitamin D or calcium.

**Conclusions:**

These data provide no evidence of association between degree of metabolic control and BMD in adolescents with T1D. Adolescents with T1D have a high prevalence of serum 25-hydroxyvitamin D abnormalities. Longitudinal studies are needed to evaluate the predictive value of vitamin D abnormalities on fracture risk.

## Introduction

The effects of improved home blood glucose monitoring, pharmacotherapy, and educational interventions have led to a longer lifespan for patients with type 1 diabetes mellitus (T1D). However, bone health remains a problem for many with T1D, as adults with T1D have increased fracture risk and generalized osteoporosis [1,2], and abnormalities in bone mineral density (BMD) have been reported in adolescents with T1D. The underlying mechanisms triggering the changes in BMD in patients with T1D are not well-known. Reports of the relationships between metabolic control, BMD, and bone marker parameters in patients with T1D have been conflicting [3,4]. Evaluations of bone disease in adults with T1D are generally complicated by the presence of other diabetic complications such as nephropathy, muscle insufficiency, or impaired vision that can affect bone disease. Early detection, prior to other diabetes complications, of derangements in bone markers can provide insight into the pathogenesis of bone disease in patients with T1D.

Bone health is dependent upon appropriate regulation of both anabolic and catabolic processes. Insulin-like growth factor I (IGF-I) is an anabolic regulator of bone metabolism, and BMD has been positively correlated with IGF-I levels in both human and animal studies [5-7]. IGF-I is decreased in patients with T1D, associated with the degree of metabolic control [8-10]. Patients with T1D have dysregulation of the growth hormone-IGF-I axis [9-11] and dysregulation of IGF binding proteins (IGFBP) [9,11], which determine the tissue availability of IGF-I. Parathyroid hormone has both anabolic and catabolic effects on bone. Low, unaltered or elevated levels of intact parathyroid hormone (iPTH) have previously been reported in patients with T1D [12,13]. However, these studies evaluated few patients and included patients with important confounders such as diabetic nephropathy that may have led to secondary hyperparathyroidism. Aberrations in bone markers may be predictive of osteoporosis and fractures [14]. Bone-specific alkaline phosphatase, which is a bone formation marker reflective of osteoblastic activity, is lower in patients with T1D than controls [15]. In addition, urinary cross linked N-telopeptides of type I collagen (NTX), bone breakdown markers, are decreased in patients with T1D [16]. Bone turnover therefore is decreased in patients with T1D, although the etiologies are unclear.

Peak bone mass is attained by early adulthood [17], and therefore interference with this process in adolescence results in life-long complications. Evaluations of bone disease in adolescents with T1D are limited. This study was designed to evaluate whether in adolescents with T1D: 1) poor metabolic control is associated with reduced BMD; 2) changes in biochemical bone parameters such as IGF-I system components, iPTH, bone turnover markers, and 25-hydroxyvitamin D are associated with metabolic control. We also hypothesized that adolescents with T1D would have a higher prevalence of vitamin D deficiency than the healthy adolescent population.

## Materials and methods

### Participants

Adolescents (ages 13-18 years) with T1D for ≥ 3 years were recruited from the pediatric clinic in the Eskind Diabetes Center at Vanderbilt Medical Center. All participants were diagnosed with T1D by a pediatric endocrinologist. The study was conducted in accordance with the Declaration of Helsinki and was approved by the Vanderbilt University Medical Center Institutional Review Board. Informed consent was obtained from each participant's parent/legal guardian, and informed assent was obtained from each participant prior to beginning the study. Participants were categorized by the degree of glucose control. Poor control (PC) was defined as all (must have a minimum of 3) HbA1c measurements ≥ 9% and favorable control (FC) was defined as all HbA1c measurements < 9% during the previous 2 years. Exclusion criteria were: presence of microalbuminuria, retinopathy or neuropathy; pre-existing bone disease, cystic fibrosis or celiac disease, eating disorder, estro-progesterone or testosterone treatment including oral contraceptives, smoking, pregnancy, amenorrhea, polycystic ovarian syndrome as diagnosed by a pediatric endocrinologist based upon irregular menses as well as hirsutism and/or biochemical evidence of androgen excess, obesity [body mass index (BMI) > 95th percentile for age and sex], short stature (< 3rd percentile for age and sex), or delayed/precocious puberty.

### Anthropometric Evaluation

Height and weight were obtained, and body mass index was calculated based on the following formula: weight (kg)/height (m)^2^.

### Response variables

#### 1. Bone measurements

The primary dependent variable was BMD measured by DEXA scan.

Total BMD, lumbar spine (L2-L4) BMD, L2-L4 width, and femoral neck BMD were determined by dual energy radiographic absorptiometry (DEXA, GE Healthcare, Lunar iDEXA, Tube model 40782). Prior to September 2007, DEXA scans were performed using the GE Healthcare Lunar Model 8743. Lumbar (L2-L4) BMD and L2-L4 width were used to determine bone mineral apparent density (BMAD, grams per cubic centimeter), which was calculated based on the following formula: BMD (L2-L4) × (4/[*pi *x width]) [18]. Results were expressed as z-scores for total body BMD, lumbar BMD, BMAD, and femoral neck BMD. Z-scores were calculated using the means and SD reported for age and gender [19]. Serum pregnancy tests were negative on all females prior to DEXA scan.

#### 2. Laboratory Evaluation

Other dependent variables included components of the IGF system, bone turnover makers, and serum 25-hydroxyvitamin D levels. Venous blood samples were collected after an overnight fast (approximately 10 hours). Participants received their usual evening insulin glargine dose the night prior or were continued on their insulin pump at the usual basal rate. For each participant, serum for IGF-I, IGFBP-3 (insulin-like growth factor binding protein 3), IGFBP-4, IGFBP-5, iPTH, bone-specific alkaline phosphatase (BAP), total 25-hydroxyvitamin D, calcium, phosphorus, and creatinine were obtained. Urine was collected for 24 hours at home prior to arrival for analysis for cross linked N-telopeptides of type I collagen (NTX), creatinine, calcium, and phosphorus.

#### 3. Diet diary

Subjects were asked to keep a diet diary for 72 hours prior to the study visit, in order to ascertain information regarding dietary vitamin D and calcium intake. Each subject was instructed to record type of food/beverage and amount consumed in as much detail as possible. Verbal and written instructions were given to each subject and his/her parents when consented for the study, and logsheets were provided for each subject. The information from these diet diaries was placed into the Nutrition Data System for Research (NDSR, University of Minnesota), and an analysis of calcium and vitamin D intake per day was obtained.

### Exposure variable

The primary independent variable wa*s *the average of 3or more consistent levels of HbA1c in the 2 years prior to enrollment. Subjects were assigned either to the poor control (PC) group (HbA1c ≥ 9%) or the favorable control group (FC) (HbA1c < 9%). Subjects who had HbA1c values in both categories during the two years prior to potential study enrollment were excluded. The enrollment plan included that an equal number (± 10%) of subjects would be enrolled in the two metabolic groups. This targeted enrollment by HBA1c was to ascertain that subjects in poor control were represented in study.

### Assay methods

HbA1c was measured by point-of-care immunoassay using the DCA Vantage Analyzer (Siemens Healthcare Diagnostics, Deerfield, IL, USA). Serum concentration of total IGFI was measured by radioimmunoassay (RIA) (ALPCO Diagnostics, Salem, NH, USA). Inter- and intra-assay coefficients of variation were 3.4-4.2% and 2.6-4.1%, respectively. Serum IGFBP-3 was measured by radioimmunoassay (RIA) using rabbit polyclonal antiserum and human recombinant IGFBP-3 as standard and tracer [20]. Serum IGFBP-4 was measured by a specific RIA using recombinant human IGFBP-4 expressed in Escherichia coli as antigen, tracer and standard [21]. Antibodies against human IGFBP-4 were developed in guinea pigs as described previously [21]. Inter-and intra-assay variations were less than 8%. There was no cross-reactivity with other IGFBPs. Serum IGFBP-5 was measured by a specific RIA using recombinant human IGFBP-5 as antigen, tracer and standard as described [22]. There was no cross-reactivity with other IGFBPs. Inter-and intra-assay variations were less than 8%. Cross-linked N-telopeptides in urine were measured by chemiluminescent immunoassay (ARUP Laboratories, Salt Lake City, UT, USA). Intact PTH was measured using the Roche Cobas electrochemiluminescence assay, BAP was measured by the immunoenzymatic assay Access Ostase, and total 25-hydroxyvitamin D was measured using Liquid Chromatography-Tandem Mass Spectrometry (LC-MS/MS) (Mayo Medical Laboratories, Rochester, MN, USA). The criteria used to define vitamin D sufficiency, insufficiency, and deficiency were 25-hydroxyvitamin D levels ≥ 30 ng/mL, 15-29 ng/mL, and <15 ng/mL, respectively, as was recently reported [23].

### Statistical methods

Clinical and demographic variables are presented as number and percent or medians and interquartile ranges [IQR]. Categorical variables were compared by HbA1c degree of control group with χ^2 ^test and continuous variables with Wilcoxon rank sum test. Spearman correlation coefficients (rho) were used to assess the correlation of continuous BMD measures, components of the IGF system, bone turnover markers, vitamin D and calcium intake, age, and HbA1c. We assessed for the adjusted association between BMD measures, components of the IGF system, bone turnover markers, vitamin D and calcium intake and age with HbA1c as an independent variable with multiple linear regression analyses. Multivariable linear regression assumptions of normality of residuals were checked and were met. Covariates for adjustment were chosen *a priori *and included age, gender and race. All reported *p *values are unadjusted for multiple tests. All analyses used a 5% two-sided significance level and were performed with R version 2.10.10 http://www.r-project.org. To compare the prevalence of vitamin D status with published NHANES study report[23], we calculated the proportion of subjects who were insufficient and deficient in 25-hydroxyvitamin D by gender and calculated the 95% confidence interval (CI) for binomial proportion using the Wilson method. The Wilson method was used as it works better for calculation of 95%CI with small samples and extreme probability (approaching 1 or 0) [24].

## Results

### Demographic characteristics

The study population included 57 participants, 30 in the FC group and 27 in the PC group (stable HbA1c during the 2 years prior to study participation). There were no significant differences between groups in age, gender, duration of diabetes, or anthropometrics (Table 1). As expected based on selection criteria, the average HbA1c during the two years prior to the study in the PC group was higher than that in the FC group.

**Table 1:** Characteristics of subjects by metabolic control, results shown as median and [IQR] interquartile range.

	Favorable Control (n = 30)	Poor Control (n = 27)	*p *value*
**HbA1C (%)**	**7.7 [7.3, 7.9]**	**10.3 [9.9, 11.4]**	**

Age (yrs)	15.2 [14.3, 16.1]	16.4 (15.1, 17.8)	0.05

Gender (% female)	37%	59%	0.42

Race (% Caucasian)	87% [26/30]	81%[21/26]	0.55

Duration DM (yrs)	6.5 [4.8,8.0]	6.5[5.5,9.5]	0.40

Height (cm)	169.8 [ 158.6,178.1]	163.2 [16.03,165.7]	0.22

Weight (kg)	68.5 [55.4,74.0]	67.5 [ 59.0,77.5]	0.91

BMI (kg/m^2^)	22.8 [20.4,27.5]	25.0 [21.4,27.9]	0.35

*Wilcoxon rank sum test for continuous variables or χ^2 ^test for categorical variables.

** Groups were defined based on HbA1c

### BMD, bone markers, and components of the IGF system in FC and PC groups

The FC group had a higher iPTH level [30.5 pg/ml (21.8, 37.8)] compared with the PC group [19 pg/ml (15, 31)], p = 0.04. The FC group also had higher urine n-telopeptides (NTX) than the PC group [238 nM BCE/mM creat (98,366) vs 56 nM BCE/mM creat (41,311), p = 0.03] and a lower urine Ca/Cr than the PC group [0.07 (0.05, 0.15) vs 0.14 (0.09, 0.21), p = 0.02], consistent with higher iPTH levels (Table 2). There were no differences between groups in measurements of BMD, IGFI or IGFBPs, BAP, serum calcium, serum creatinine, eGFR, or 25-hydroxyvitamin D levels.

**Table 2:** Clinical and biochemical features of subjects with T1D

	n	FC (n = 30)	PC (n = 27)	*p *value†
Lumbar spine BMD*	56	0.01 (-1.14, 1.07)	-0.46 (-0.82, 0.37)	0.58
Volumetric lumbar spine (BMAD)*	56	0.05 (-0.63, 0.69)	-0.14 (-0.48, 0.57)	0.83
Femoral neck BMD*	56	0.12 (-1.23, 0.98)	-0.48 (-1.19, 1.39)	0.66
Total body BMD*	56	0.46 (-1.27, 1.35)	-0.03 (-1.1, 0.72)	0.66
% body fat	56	28.7 (16.7, 35.3)	33.7 (20.2, 40)	0.22
IGF-1 (ng/mL)	56	564 (430, 644)	488 (402, 603)	0.29
IGF BP-3 (μg/L)	54	2883 (2448, 3060)	2901 (2676, 3436)	0.36
IGF BP-4 (μg/L)	54	398.5 (334.8, 439.2)	415.5 ± 57.9	0.17
IGF BP-5 (μg/L)	54	346.0 (300.8, 384.2)	316.5 (273.2, 447.8)	0.55
**NTX (nM BCE/mM creat)**	**54**	**238 (98, 366)**	**56 (41, 311)**	**0.03**
Bone-Specific Alkaline Phosphatase (ug/L)	53	34 (23, 63)	19 (14.5, 49.5)	0.09
**Intact PTH (ρg/mL)**	**53**	**30.5 (21.8, 37.8)**	**19 (15, 31)**	**0.04**
Serum 25-hydroxyvitamin D (ng/mL)	52	31 (21, 37)	32 (24, 38.5)	0.76
Serum Calcium (mg/dL)	56	9.4 (9.3, 9.7)	9.4 (9.2, 9.6)	0.99
Serum Phosphorus (mg/dL)	56	4.4 (4.1, 5.0)	4.8 (4.3, 5.2)	0.21
Serum creatinine (mg/dL)	56	0.75 (0.63, 0.8)	0.7 (0.6, 0.8)	0.22
eGFR estimation (ml/min)	56	137.5 (122.7, 149.8)	141.8 (118.2, 166.4)	0.72
**uCa/uCr**	**54**	**0.07[0.05, 0.15]**	**0.14[0.09,0.21]**	**0.02**
uPhos/uCr	54	0.80[0.64, 0.92]	0.90[0.72, 1.0]	0.22
Average vitamin D intake/day (IU)	25	173 (128, 246)	168 (122, 246)	1
Average calcium intake/day (mg)	25	872 (651, 1136)	686 (574, 927)	0.46

Items in bold p < 0.05.

† Wilcoxon rank sum test for comparison of continuous variables

* z-scores

Abbreviations: FC: Favorable Control, PC: Poor Control, BMD: bone mineral density, BMAD: bone mineral apparent density, eGFR (estimated glomerular filtration rate)

### Relationship between metabolic control and BMD, bone markers, IGF system components and vitamin D/calcium intake

Univariate analyses (Table 3) demonstrated a positive correlation between HbA1c and % body fat (rho = 0.30, p = 0.02) and urine Ca/Cr (rho = 0.27, p = 0.047) and negative correlations between HbA1c and NTX (rho = -0.36, p = 0.01), intact PTH (rho = -0.28, p = 0.04), and serum creatinine (rho =-0.27, p = 0.04). There were no other significant correlations between HbA1c and any of the other study variables. After controlling for age, sex, and race, there was a positive association between HbA1c and serum phosphorus and a negative association between HbA1c and serum creatinine that were statistically significant. There were no associations between HbA1c and BMD, bone turnover markers, components of the IGF system, serum 25-hydroxyvitamin D, serum calcium, or dietary intake of calcium and vitamin D.

**Table 3:** Association between HbA1c and bone mineral density measurements, components of the IGF system, bone turnover markers, and dietary intake of calcium and vitamin D

	Spearman correlation coefficient	p value*	Adjusted p value **
Age	0.24	0.08	

Lumbar Spine BMD †	-0.12	0.37	0.63

Volumetric Lumbar Spine † (BMAD)	-0.041	0.76	0.85

Femoral neck BMD †	-0.056	0.68	0.24

Total body BMD †	-0.086	0.53	0.87

**% Body fat **	**0.30**	**0.02**	0.66

IGF-I (ng/ml)	-0.15	0.27	0.92

IGFBP3 (μg/L)	0.13	0.37	0.33

IGFBP4 (μg/L)	0.23	0.10	0.1

IGFBP5 (μg/L)	-0.09	0.52	0.65

**NTX (nM BCE/mM creat)**	**-0.36**	**0.01**	0.73

Bone-specific alkaline phosphatase (μg/L)	-0.26	0.06	0.81

**Intact PTH (ρg/ml)**	**-0.28**	**0.04**	0.2

Serum 25-hydroxyvitamin D (ng/ml)	-0.047	0.74	0.89

Serum Calcium (ml/dL)	0.15	0.28	0.19

Serum Phosphorus (mg/dL)	0.2	0.14	**0.01**

**Serum creatinine (mg/dL)**	**-0.27**	**0.04**	**0.007**

**UCa/uCr**	**0.27**	**0.047**	**0.05**

uPhos/uCr	0.22	0.1	0.37

Average vitamin D intake/day (IU)	-0.09	0.67	0.55

Average calcium intake/day (mg)	-0.13	0.55	0.42

Items in bold p < 0.05.

*Spearman correlation coefficient p value; ** p value adjusted for age, race, and gender; † z-scores

### Dietary Intake of vitamin D and calcium

Subjects with T1D who have favorable metabolic control report consuming a similar amount of vitamin D and calcium as those who have poor metabolic control (Table 2). Only 12% (3/25) of the total subgroup with intake data reported consuming the recommended daily allowance (RDA) of vitamin D (400 IU/day at the time of the study), and 16% (4/25) of the total subgroup reported consuming the RDA for calcium (1300 mg/day), with no differences between groups (18% vs 12%, p = 0.74). When evaluated with the Institute of Medicine's 2011 RDA of 600 IU vitamin D/day [25], only 1 subject reported consuming this amount.

*Hydroxyvitamin D status*There were no differences between groups in serum 25-hydroxyvitamin D levels (Table 2), with 14% of the FC group deficient (25-hydroxyvitamin D levels < 15 ng/ml) and 31% insufficient (25-hydroxyvitamin D levels 15-29 ng/ml) and 13% of the PC group deficient and 35% insufficient in serum 25-hydroxyvitamin D, p = 0.96. Together, there were no differences between subjects with T1D and the normal adolescent population [23] in the prevalence of 25-hydroxyvitamin D deficiency, as 11% (95%CI: 2.9%, 31.4%) of girls and 0% (95%CI: 0, 13.3%) of boys with T1D were deficient in 25-hydroxyvitamin D, compared with 5% and 3% of the normal adolescent population, respectively. Thirty-two percent of girls (95%CI: 15.4%, 54.0%) and 40% (95%CI: 23.4%, 59.2%) of boys were insufficient in 25-hydroxyvitamin D, compared with 57% and 63%, respectively, of the general adolescent population. When using the 2011 Institute of Medicine Report's definition of vitamin D inadequacy [25], 21% of the FC group and 17% of the PC group had 25-hydroxyvitamin D levels <20 ng/ml, p = 0.76.

## Discussion

This study addresses whether metabolic control has an effect on bone health in patients with T1D who have not yet achieved peak bone mass. Adolescents with T1D in consistently poor metabolic control appear to have lower iPTH levels than a group of adolescents with T1D in favorable metabolic control, although this association was attenuated when adjusted for age, gender and race. After adjustment, urine calcium is associated with metabolic control, consistent with lower iPTH levels. We also explored the prevalence of 25-hydroxyvitamin D abnormalities in adolescents with T1D, as vitamin D abnormalities have been increasingly recognized as a significant health problem both for healthy patients and those with chronic illnesses [26]. Adolescents with T1D have significant 25-hydroxyvitamin D abnormalities, but these abnormalities are not more prevalent than in the general adolescent population.

Poor bone health is a significant problem for many adults with T1D, as is demonstrated by a two-fold increase in fracture risk in the lumbar spine, femoral neck, and distal radius [1]. Women with T1D have more than a 10-fold increase in risk of hip fractures compared with age-matched controls [2]. Almost 20% of patients with T1D ages 20-56 years meet criteria for osteoporosis [27], which is a debilitating illness that impairs functionality and quality of life [28]. However, the etiologies of decreased BMD in adults with T1D have been unclear [29,30]. The mechanisms of osteoporosis associated with T1D differ from the development of osteoporosis associated with aging [31], and several potential mechanisms have been proposed, including effects of advanced glycation end products in bone collagen [32], increased urinary excretion of calcium, phosphate and magnesium [33], inflammatory cytokines [34], low levels of iPTH [12], diabetic microangiopathy with reduced blood flow to bone [35], decreased bone resorption [16], decreased bone formation [36], and vitamin D deficiency [37]. We sought to evaluate whether poor metabolic control during adolescence is associated with abnormal bone health.

Previous studies investigating the role of metabolic control and BMD in patients with T1D have been limited by older BMD measurement methods which may be inaccurate, limited knowledge in the consistency of patients' actual metabolic control (many studies use only a single HbA1c measurement as the index of metabolic control), and confounding factors affecting BMD such as the use of oral contraceptives. In this study, we controlled confounding factors and found no correlations between metabolic control and BMD, similar to others [3,38,39], but differing from some [4]. Although subjects had stable diabetes control for two years prior to the study, two years of poor diabetes control may not be long enough to lead to changes in BMD demonstrated by DEXA scans in adolescents.

The role of IGF-I as a critical anabolic regulator of BMD is clearly demonstrated in animal studies in which genetic manipulation of the IGF system led to osteopenia [7]. In humans, cross-sectional and cohort studies in various populations, including subjects from the Framingham Heart Study, have demonstrated a strong correlation between serum levels of IGF-I and BMD [6,40]. Dysregulation of the IGF-I/IGFBP system has been reported in patients with T1D [9-12,32]. We found no associations between metabolic control and components of the IGFI system, although other studies have reported that IGF-I levels correlate with metabolic control [11]. As we did not have a control group of healthy subjects, IGFI levels may be too low within a population of T1D patients to detect significant differences.

Only 35% of healthy non-Hispanic white adolescents are sufficient in 25-hydroxyvitamin D [23], which plays an important role in the maintenance of bone health. Similar to recent studies, we found a significant prevalence of 25-hydroxyvitamin D abnormalities in adolescents with T1D [37,41], but we did not find a difference in 25-hydroxyvitamin D status between adolescents with T1D and the normal adolescent population [23]. However, the proportions of vitamin D sufficiency status by gender were limited by small numbers which resulted in proportions with wide confidence intervals. There is not enough evidence to support that metabolic control plays a role in vitamin D status, and other risk factors need to be evaluated.

Only one subject reported consuming the current RDA for vitamin D, with 16% consuming the RDA for calcium. This is not different from the healthy adolescent population, as previous data from NHANES III (1988-1994) demonstrated that only 53-63% of all US children consume at least 200 IU of vitamin D per day from diet and/or supplementation. (200 IU was reported in this paper because it was considered Adequate Intake for vitamin D at that time) [42]. This is of particular concern for adolescent patients with T1D, as vitamin D and calcium intake are modifiable factors that have a significant impact on BMD.

Our study had several limitations. We stratified by design the subjects into poor and favorable metabolic control groups, and we could have missed relationships between HbA1c and study variables due to a lack of a full continuous spectrum of HbA1c values. We did not have a control group of non-diabetic patients. Also, we did not obtain information regarding exercise and specifics regarding pubertal development, which clearly have an effect on BMD and bone turnover. However, we attempted to address this issue by excluding patients with obesity as well as known pubertal delay, amenorrhea, or polycystic ovarian syndrome.

In summary, metabolic control is not associated with BMD as evaluated by DEXA in this adolescent population with T1D, and bone anabolic and turnover markers are not affected by metabolic control, once age, gender, and race are taken into account. Adolescents with T1D are frequently vitamin D insufficient, which is likely also playing an important role in bone metabolism.

## Abbreviations

T1D: type 1 diabetes mellitus; HbA1c: hemoglobin A1c; PC: poor control; FC: favorable control; NTX: urinary cross linked N-telopeptides of type I collagen; BAP: bone-specific alkaline phosphatase; IGF-I (insulin-like growth factor I).

## Competing interests

The authors declare that they have no competing interests.

## Authors' contributions

JS made substantial contributions to study design, data acquisition, analysis and interpretation of data, and drafted the manuscript. MR, KN, RH, and AS contributed substantially to study design, subject recruitment, data acquisition, and analysis and interpretation of the data. TG contributed through statistical analysis and interpretation of the data. SM contributed through study design and data acquisition. All authors critically revised the manuscript for important intellectual content and approved the final version of the manuscript.
